# Photobiomodulation (PBM) irradiation enhances the therapeutic potential of hMSC spheroids for neural repair

**DOI:** 10.3389/fncel.2026.1728579

**Published:** 2026-01-29

**Authors:** So-Young Chang, Namgue Hong, Ji Eun Choi, Jin-Chul Ahn, Min Young Lee

**Affiliations:** 1 Beckman Laser Institute Korea, Dankook University, Cheonan, Republic of Korea; 2 Medical Laser Research Center, College of Medicine, Dankook University, Cheonan, Republic of Korea; 3 Department of Otolaryngology-Head & Neck Surgery, College of Medicine, Dankook University, Cheonan, Republic of Korea

**Keywords:** hydrogen peroxide, mesenchymal stem cells, neural regeneration, oxidative stress, photobiomodulation, spheroids

## Abstract

Neural regeneration remains a critical goal in regenerative medicine, especially for treating central nervous system injuries such as stroke, spinal cord injury, and neurodegenerative diseases. Mesenchymal stem cells (MSCs) have shown therapeutic potential through their capacity for differentiation and paracrine signaling; however, their clinical application is limited by low survival and engraftment rates. In this study, we investigated whether the therapeutic efficacy of human MSC (hMSC) spheroids could be enhanced through photobiomodulation (PBM). hMSCs were aggregated into three-dimensional spheroids and divided into four experimental groups: (1) untreated control spheroids, (2) spheroids treated with 660 nm PBM, (3) spheroids treated with 850 nm PBM, and (4) spheroids co-cultured with primary rat cortical neurons subjected to oxidative stress using hydrogen peroxide (H₂O₂). The PBM groups were exposed to red (660 nm) or near-infrared (NIR; 850 nm) light for 10 min. Neuronal viability and axonal regeneration were assessed. Our results demonstrated that PBM-treated hMSC spheroids significantly increased neuronal survival and axonal outgrowth compared to H₂O₂-only controls, particularly under high oxidative stress conditions. Notably, spheroids treated with 850 nm PBM exhibited the most robust neuroprotective effects. These findings suggest that PBM enhances mitochondrial activity and the secretion of neurotrophic factors by hMSC spheroids, thereby promoting neuroregeneration. This combinatorial strategy integrating PBM with 3D stem cell spheroid culture offers a promising avenue for developing advanced stem cell therapies for neurological disorders.

## Introduction

Neural regeneration remains a critical goal in the field of regenerative medicine, with the potential to treat a wide range of neurological disorders such as peripheral nerve injuries, spinal cord injuries, stroke, Parkinson’s disease, and traumatic brain injuries (Kvistad et al., 2024; Zhu et al., 2025; Li et al., 2023; Andrzejewska et al., 2021; Yarahmadi, 2025; Rao et al., 2022; Kim and Kwak, 2025; Arslan et al., 2024). Achieving effective repair of neural structures requires innovative approaches that can stimulate the body’s inherent healing capabilities. Traditional therapeutic strategies for these disorders, such as pharmacological agents, surgery, and physical rehabilitation, often fall short of restoring damaged neural tissues or achieving sustained functional recovery, thereby highlighting the urgent need for more effective regenerative approaches (Carter et al., 2019). Among these approaches, stem cell-based therapies have gained significant attention due to their ability to replace damaged cells and modulate the local microenvironment through paracrine signaling (Wang et al., 2025; Zeng, 2023; Zhidu et al., 2024; Xue et al., 2024; Carpena et al., 2025; Chang, 2023; Chang and Lee, 2021; Abueva et al., 2025).

Stem cells—particularly mesenchymal stem cells (MSCs)—are capable of differentiating into various cell types, including neurons, glial cells, and other supportive tissues (George et al., 2019; Xu et al., 2024). Beyond differentiation, they secrete neurotrophic factors such as the brain-derived neurotrophic factor (BDNF), vascular endothelial growth factor (VEGF), and other cytokines, which promote neuroprotection, angiogenesis, and tissue repair (Isakovic, 2023). For example, MSCs have been utilized in experimental models to improve functional recovery after spinal cord injury by reducing inflammation and promoting axonal growth. Nevertheless, their therapeutic potential in clinical settings is often hampered by poor cell survival, limited engraftment, and inconsistent functional outcomes following transplantation (Kusuma, 2022).

The three-dimensional organization of stem cells into spheroids offers several key advantages over traditional monolayer cultures or dispersed single cells (Petrenko et al., 2017; Yen et al., 2023). Spheroid formation enhances cell–cell and cell–extracellular matrix (ECM) interactions, increases survival rates, and amplifies the secretion of therapeutic factors (Yen et al., 2023; Xu, 2016). For instance, MSC spheroids have demonstrated enhanced secretion of neurotrophic factors compared to single-cell suspensions, leading to improved outcomes in laboratory models of nerve injury. This 3D configuration also more closely mimics the *in vivo* microenvironment, providing structural and biochemical cues essential for maintaining stem cell potency and function (Jaukovic, 2020).

Photobiomodulation (PBM), a non-invasive light therapy, has also been shown to bolster cellular activity and enhance the therapeutic potential of stem cells (Chang, 2023; Chang and Lee, 2021; Abueva et al., 2025; Chang, 2020; Park, 2022; Hong et al., 2022). PBM is most commonly applied within the red to near-infrared (NIR) spectrum, typically ranging from 600 to 1,000 nm, due to its favorable tissue penetration and interaction with mitochondrial photoacceptors, such as cytochrome c oxidase (Zhang et al., 2024; Fernandes et al., 2024).

PBM acts primarily by stimulating mitochondrial cytochrome c oxidase, thereby increasing ATP production, modulating reactive oxygen species, and improving cell proliferation, differentiation, and survival (Hamblin, 2018; Chang et al., 2019).

Recent systematic reviews have highlighted that PBM within this wavelength range plays an important role in modulating neural signaling pathways and cellular bioenergetics, underscoring the importance of wavelength selection and parameter optimization for neural repair and other biomedical applications (Zhang et al., 2024; Fernandes et al., 2024). Within this wavelength range, PBM has been widely investigated for various biomedical applications, including neural repair, wound healing, and inflammation control. These effects are particularly beneficial for MSCs, as PBM has also been reported to rejuvenate aged or stressed cells by restoring mitochondrial function and boosting paracrine signaling (Kusuma, 2022; Hung et al., 2022).

When combined with MSC spheroids, PBM can further amplify their regenerative effects, making this a promising strategy for neural repair (Mulaudzi et al., 2024). However, most previous PBM studies have been conducted in monoculture systems or simple two-dimensional (2D) environments, which do not fully capture the complex cellular interactions involved in neural repair. Therefore, optimizing the activity and functionality of MSC spheroids prior to clinical application remains essential. Preconditioning strategies, such as PBM, can enhance therapeutic efficacy, leading to better integration, survival, and functional recovery in injured neural tissues. Improving the potency of MSC spheroids through such methods is a vital step toward translating these promising therapies from laboratory research to real-world clinical treatments.

In this study, we explored the regenerative potential of human MSC (hMSC) spheroids preconditioned with PBM at different wavelengths in a hybrid co-culture system. Unlike previous studies that primarily focused on PBM effects in single-cell populations or homogeneous culture models, our approach integrated three-dimensional hMSC spheroids with a primary rat cortical neuron monolayer, enabling the investigation of PBM-mediated neuron–stem cell interactions in a spatially organized *in vitro* environment. These spheroids were co-cultured with primary rat cortical neurons that had been damaged by hydrogen peroxide (H₂O₂)-induced oxidative stress, a model that mimics aspects of neural injury and degeneration.

## Materials and methods

### Animals

All experimental procedures were reviewed and approved by the Institutional Animal Care and Use Committee (IACUC) of Dankook University Medical School (approval number: DKU-23-058) and were conducted in accordance with the National Institutes of Health (NIH) guidelines for the care and use of laboratory animals. Furthermore, we confirm that all procedures are reported in compliance with the ARRIVE guidelines. Animals were housed under a 12-h light/dark cycle with free access to standard food pellets and water.

### Primary culture of cortical neurons

Primary cortical neurons were prepared from embryonic day 17 (E17) Sprague–Dawley rat fetuses, as previously described with minor modifications (Hong et al., 2023). Pregnant rats were anesthetized with 16.5% urethane, and the fetal cortices were dissected in calcium- and magnesium-free HEPES-buffered Hank’s balanced salt solution (pH 7.45, Gibco, Life Technologies Corporation, NY, United States). Cortical tissues were gently dissociated using a series of flame-polished Pasteur pipettes. The dissociated cells were seeded at a density of 1.6 × 10^4^ cells/well onto 25-mm glass coverslips pre-coated with 0.2 mg/mL Matrigel (Corning, Bedford, MA, United States) for 1 h. Neurons were maintained in Neurobasal medium supplemented with 2% B27 (Gibco; Thermo Fisher Scientific, Inc., Waltham, MA, United States), 0.25% GlutaMAX-I(Gibco; Life Technologies Corporation), 100 U/mL penicillin, 100 μg/mL streptomycin, and 0.025 μg/mL amphotericin B (Sigma-Aldrich, St. Louis, MO, United States) in a humidified incubator (10% CO₂, 90% air, 37 °C, pH 7.4). The medium was replenished every 4 days by replacing 75% of the volume with fresh medium. All experiments were conducted using six independent cultures. On day *in vitro* (DIV) 13, neurons were exposed to either 10 μM or 100 μM hydrogen peroxide (H₂O₂) for 30 min, after which the medium was completely replaced with fresh culture medium.

### Human mesenchymal stem cell isolation, culture, and spheroid production

This study was approved by the Institutional Review Board of Dankook University Hospital (Approval No. DKUH-2020-05-001), and all procedures were conducted in accordance with the Declaration of Helsinki. Informed consent was obtained from all donors prior to the collection of human-derived biological materials (hMSCs). The manuscript does not contain any personally identifiable information of the donors other than age and sex. A 50-year-old female patient who provided informed consent prior to surgery participated in this study. Cell sorting and differentiation were performed by N-BIOTEK (Bucheon-si, Gyeonggi-do, 14,502, Republic of Korea), which is an officially approved stem cell engineering center by the Korean Government. Further information is provided in a previous publication (Chang et al., 2023). hMSCs were isolated from human adipose tissue. The adipose tissue was washed three times with 1X phosphate-buffered saline (PBS) and digested with 0.1% (w/v) type I collagenase (Nordmark, Uetersen, Germany) for 45 min at 37 °C. Undigested tissue and excess oil were filtered out using a screen mesh, followed by centrifugation at 1500 rpm for 5 min. The passage number used in the experiment was passage 4. hMSCs were cultured in *α*-MEM (Corning) supplemented with 10% fetal bovine serum (FBS, Gibco) and 1% penicillin–streptomycin at 37 °C in a 5% CO₂ incubator. To confirm the characteristics of the hMSC spheroids, the expression of CD73 (Invitrogen, 41–0200), CD90 (Abcam, ab307736), and CD105 (Invitrogen, MA5-17041) was examined as a positive marker, while CD45 (Abcam, ab10558) was examined as a negative marker. Cells were seeded in 90 mm culture dishes and subcultured when they reached approximately 80–90% confluence. For spheroid formation, hMSCs were seeded in ultra-low attachment U-bottom 96-well plates (Sumitomo Bakelite Co., Ltd., Kobe, Japan) at a density of 3.5 × 10^3^ cells per well in 100 μL of complete medium. The plates were centrifuged at 1,000 rpm for 3 min to promote cell aggregation and then incubated for 48 h to allow spontaneous spheroid formation. At the time point when primary cortical neurons had established mature neuronal networks and hMSC spheroids had developed a compact and transferable structure, the two cell types were co-cultured. One hMSC spheroid was added to each well containing cortical neurons.

### Photobiomodulation and hMSC spheroid treatment

hMSC spheroids were irradiated with either 660 nm (10 mW/cm^2^) or 850 nm (12 mW/cm^2^) wavelength light for 10 min prior to their application to cortical neuron cultures. On DIV 13, 30 min after H₂O₂ exposure, the irradiated hMSC spheroids were introduced to the neuronal cultures. PBM exposure was performed in the dark, and a power meter (PD300 and VEGA, Ophir Photonics) was used to confirm light intensity. Neurons were fixed 24 h after H₂O₂ treatment for subsequent analyses. To assess power density-dependent effects of PBM on spheroid-mediated neuroprotection, additional spheroid groups were irradiated at multiple power densities prior to co-culture. For 660 nm PBM, spheroids were exposed at 5, 10, or 20 mW/cm^2^, and for 850 nm PBM at 6, 12, or 24 mW/cm^2^ (10 min), followed by co-culture under H₂O₂ injury conditions and MTT assessment at 24 h (Supplementary Figure 1).

### Immunocytochemistry

Cortical neurons and hMSC spheroids were fixed with cold methanol (−20 °C) for 8 min. After fixation, cells were permeabilized with 0.3% Triton X-100 for 5 min and blocked with 10% bovine serum albumin (BSA) for 1 h. Neuronal samples were incubated overnight at 4 °C with a primary antibody against mouse anti-MAP2 (Sigma-Aldrich, M9942), followed by incubation with an Alexa Fluor 555-conjugated anti-mouse IgG secondary antibody (ThermoFisher, A21422). For the immunophenotypic characterization of hMSC spheroids, the samples were stained with primary antibodies against CD73 (Invitrogen, 41–0200), CD90 (Abcam, ab307736), CD105 (Invitrogen, MA5-17041), and CD45 (Abcam, ab10558), followed by appropriate Alexa Fluor 488-conjugated secondary antibodies. After staining, all samples were mounted with VECTASHIELD Antifade Medium containing DAPI (Vector Laboratories, H-1200) and imaged using a confocal microscope (FV3000, Olympus, Tokyo, Japan). Excitation/emission wavelengths for Alexa Fluor 488 and 555 were 488/520 nm and 561/568 nm, respectively.

### Microscopic imaging and quantification

Confocal imaging was performed using a 20 × objective on an FV3000 microscope (Olympus). Z-stacked images were acquired across 10 μm (1 μm step size) and merged into a single maximum intensity projection. For each condition, 3–4 random fields were captured. MAP2-labeled dendritic structures were visualized and analyzed using the Metamorph software (Molecular Devices) to assess dendritic morphology in primary cortical neurons.

### MTT and BDNF ELISA assay

To evaluate cell viability, cortical neurons were treated with H₂O₂ and hMSC spheroids. After 24 h, cells were incubated for 4 h with 0.5 mg/mL 3-(4,5-dimethylthiazol-2-yl)-2,5-diphenyltetrazolium bromide (MTT, Sigma-Aldrich). The resulting formazan crystals were dissolved in dimethyl sulfoxide, and absorbance was measured at 570 nm using a microplate reader. The levels of neurotrophic and angiogenic factors secreted by hMSC spheroids were quantified using enzyme-linked immunosorbent assays (ELISA). Brain-derived neurotrophic factor (BDNF), nerve growth factor (NGF), and vascular endothelial growth factor (VEGF) concentrations in culture supernatants were measured using the following commercially available human ELISA kits: Human BDNF SimpleStep ELISA Kit (ab212166, Abcam, United Kingdom), Human NGF ELISA Kit (ab193760, Abcam, United Kingdom), and Human VEGF ELISA Kit (ab222510, Abcam, United Kingdom), according to the manufacturers’ instructions. Briefly, culture media were collected immediately after the PBM treatment of hMSC spheroids, centrifuged at 2,000 × g for 5 min to remove cellular debris, and stored at −20 °C until analysis. For each assay, standards and samples (50 μL per well) were added to pre-coated 96-well plates, followed by the addition of the corresponding antibody cocktail. The plates were incubated for 1 h at room temperature with gentle shaking. After washing, tetramethylbenzidine (TMB) substrate solution was added, and the reaction was stopped using stop solution. Absorbance was measured at 450 nm using a microplate reader. All samples were analyzed in duplicate, and concentrations were calculated based on standard curves generated for each factor.

### Statistical analysis

The results were analyzed using the Prism software 8.4.3 (GraphPad Software, La Jolla, CA, United States; RRID: SCR_002798). Normality of the data distribution was assessed prior to statistical analysis. Two-tailed Mann–Whitney U tests (non-parametric) and unpaired non-parametric *t*-tests were performed to compare the hMSC size over time, as these datasets did not meet the assumptions of normality and involved independent group comparisons. One-way repeated measure analysis of variance (ANOVA) followed by Tukey’s multiple comparisons tests was used to compare cell viability, axonal length, and branch number, as these parameters were measured repeatedly across multiple experimental conditions within the same samples and met parametric assumptions. *p*-values < 0.05 were considered to indicate significance. All statistical comparisons and corresponding *p*-values for each figure are summarized in Table 1.

**Table 1:** Summary of statistical analyses and *p*-values corresponding to each figure.

Figure	Test type	Comparison	*p*-value
Figure 1D	Unpaired *t*-test		***p = 0.0001
Figure 1F	Unpaired *t*-test		***p < 0.001
Figure 2A	One-way ANOVA		***p = 0.0004
Figure 2A	Tukey’s multiple comparisons	Control vs. H_2_O_2_ (10 μM)	***p = 0.0016
Figure 2B	One-way ANOVA		****p < 0.0001
Figure 2B	Tukey’s multiple comparisons	Control vs. H_2_O_2_ (100 μM)	****p < 0.0001
Figure 2B	Tukey’s multiple comparisons	H_2_O_2_ (100 μM) vs. H_2_O_2_ (100 μM) + SP + 660 nm	***p = 0.0006
Figure 2B	Tukey’s multiple comparisons	H_2_O_2_ (100 μM) vs. H_2_O_2_ (100 μM) + SP + 850 nm	**p = 0.0013
Figure 2B	Tukey’s multiple comparisons	H_2_O_2_ (100 μM) + SP vs. H_2_O_2_ (100 μM) + SP + 660 nm	**p = 0.0066
Figure 2B	Tukey’s multiple comparisons	H_2_O_2_ (100 μM) + SP vs. H_2_O_2_ (100 μM) + SP + 850 nm	*p = 0.0148
Figure 3D	One-way ANOVA		****p < 0.0001
Figure 3D	Tukey’s multiple comparisons	Control vs. H_2_O_2_ (10 μM)	**p = 0.0015
Figure 3D	Tukey’s multiple comparisons	Control vs. H_2_O_2_ (100 μM)	**p = 0.0023
Figure 3D	Tukey’s multiple comparisons	H_2_O_2_ (100 μM) vs. H_2_O_2_ (100 μM) + SP + 660 nm	*p = 0.0148
Figure 3D	Tukey’s multiple comparisons	H_2_O_2_ (100 μM) vs. H_2_O_2_ (100 μM) + SP + 850 nm	**p = 0.0083
Figure 3E	One-way ANOVA		****p < 0.0001
Figure 3E	Tukey’s multiple comparisons	Control vs. H_2_O_2_ (10 μM)	***p = 0.0008
Figure 3E	Tukey’s multiple comparisons	Control vs. H_2_O_2_ (100 μM)	**p = 0.0019
Figure 3E	Tukey’s multiple comparisons	H_2_O_2_ (10 μM) vs. H_2_O_2_ (10 μM) + SP + 850 nm	*p = 0.0263
Figure 3E	Tukey’s multiple comparisons	H_2_O_2_ (100 μM) vs. H_2_O_2_ (100 μM) + SP + 660 nm	*p = 0.0122
Figure 3E	Tukey’s multiple comparisons	H_2_O_2_ (100 μM) vs. H_2_O_2_ (100 μM) + SP + 850 nm	**p = 0.0102

This table presents the statistical tests performed, the group comparisons made, and the resulting *p*-values associated with the data shown in the figures. *Statistically significant.

## Results

### Characteristics of the primary cortical neurons and hMSCs

**Figure 1:**
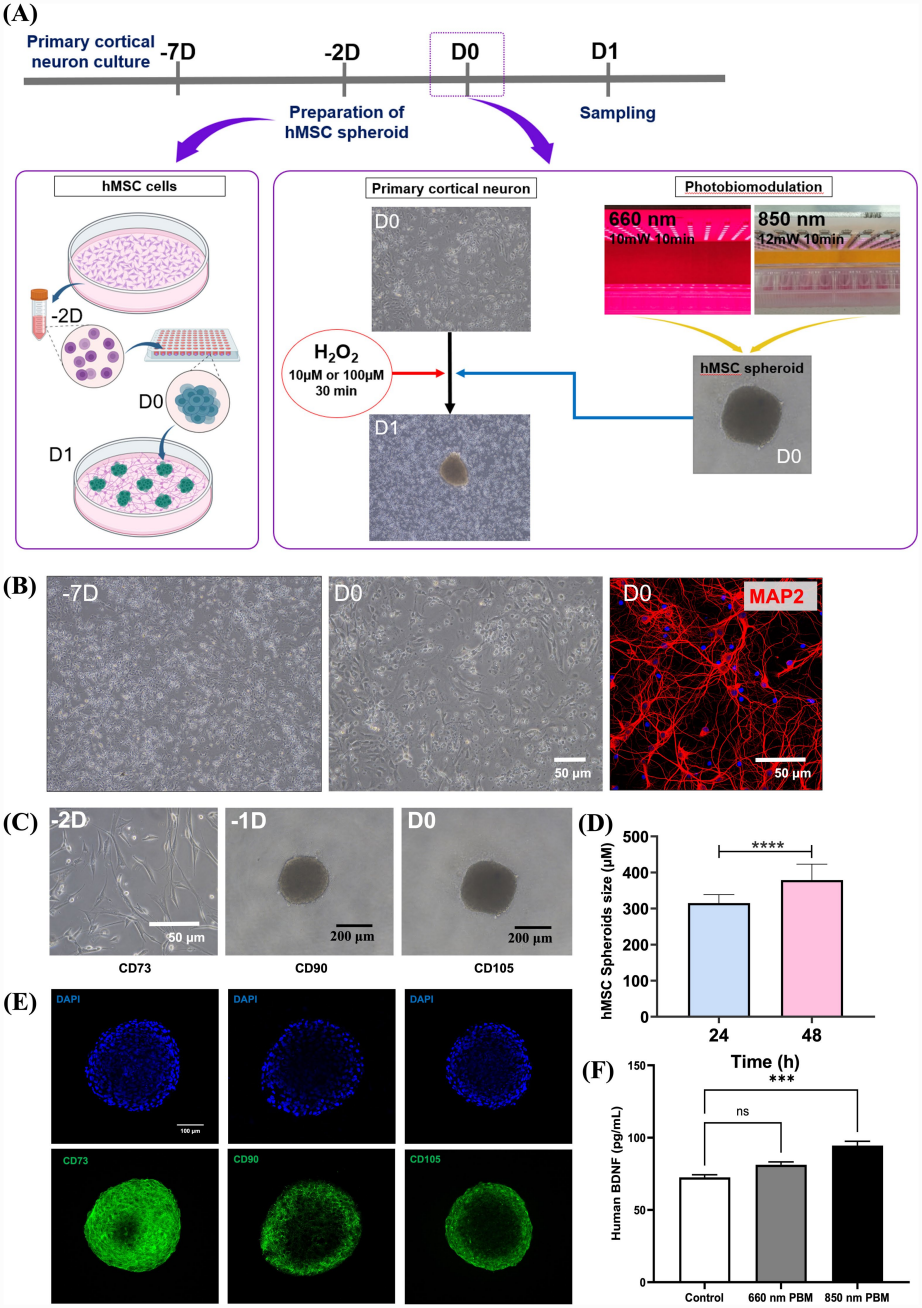
Experimental strategies using primary cortical neuron culture and photobiomodulation (PBM)-irradiated hMSC spheroids. **(A)** Primary cortical neurons were harvested from rat embryos at embryonic day 17 (E17) and cultured under conditions that promote axonal sprouting and cell–cell network formation (day −7 to day 1). In parallel, hMSCs were seeded in round-bottom 96-well plates to generate spheroids (day −2). On day 0, two-thirds of the hMSC spheroids were irradiated with photobiomodulation (PBM) at either 660 nm (10 mW, 10 min) or 850 nm (12 mW, 10 min), while the remaining spheroids were left unexposed. Primary cortical neurons were then exposed to cytotoxic H₂O₂ (10 or 100 μM for 30 min) and subsequently co-cultured with hMSC spheroids with or without PBM treatment. Samples were collected on day 1 for cell viability and epifluorescence analyses. **(B)** Immediately after seeding, cortical cells exhibited heterogeneous morphology with high cell density (day −7). Following cultivation under neural differentiation conditions, cell density decreased and cells displayed more homogeneous neuronal morphology. MAP2 immunostaining revealed extensive axonal network formation on day 0. **(C)** hMSCs exhibited spindle-shaped morphology prior to spheroid aggregation. **(D)** Following spheroid formation, the spheroid diameter increased over time, showing a statistically significant enlargement at 48 h compared to 24 h after initial formation (****p < 0.0001). **(E)** Characterization of hMSC spheroids by immunocytochemical staining for representative surface markers CD73, CD90, and CD105. **(F)** Quantification of BDNF levels in hMSC spheroids following PBM treatment (***p < 0.001).

To resemble regenerating neurons from various types of neuronal damage, primary cortical neurons were harvested from rat pups and cultured (Figure 1A). With the appropriate application of factors (see methods), the initially heterogeneous cell population gradually became homotypic, consisting of neural cells with axonal network connections (Figure 1B). These axonal connections were identified through epifluorescence analysis of MAP2 antibody expression. hMSCs were aggregated into spheroids (using the cell number of 3.5 × 10^3^), and these spheroids were structurally stable for over 48 h (Figure 1C). The average size of the hMSC spheroid reached 300 μm 48 h after initial cell seeding for spheroid formation, showing a statistically significant increase compared to 24 h (*p* < 0.0001; Figure 1D). To verify the characteristics of hMSC spheroids, the expression of representative hMSC markers was examined. Among these, the positive markers CD73, CD90, and CD105 were expressed (Figure 1E). The hMSC spheroids were divided into three groups: a spheroid-only group that received no additional treatment, a 660 nm PBM group irradiated for 10 min, and an 850 nm PBM group irradiated for 10 min. After this short PBM preconditioning, the hMSC spheroids were co-cultivated with primary cortical neuronal networks that had been damaged with varying concentrations of H_2_O_2_ (10 or 100 μM for 30 min; Figure 1A). A quantitative comparison of secreted neurotrophic and angiogenic factors in hMSC spheroids after PBM treatment was conducted. ELISA analysis revealed that PBM-treated hMSC spheroids exhibited significantly higher levels of the BDNF, NGF, and VEGF compared to non-PBM-treated spheroids (Figure 1F), indicating an enhanced paracrine secretory profile following PBM preconditioning.

### Cell viability alterations in response to hMSC spheroids, with or without PBM treatment, under H_2_O_2_-induced toxicity

**Figure 2:**
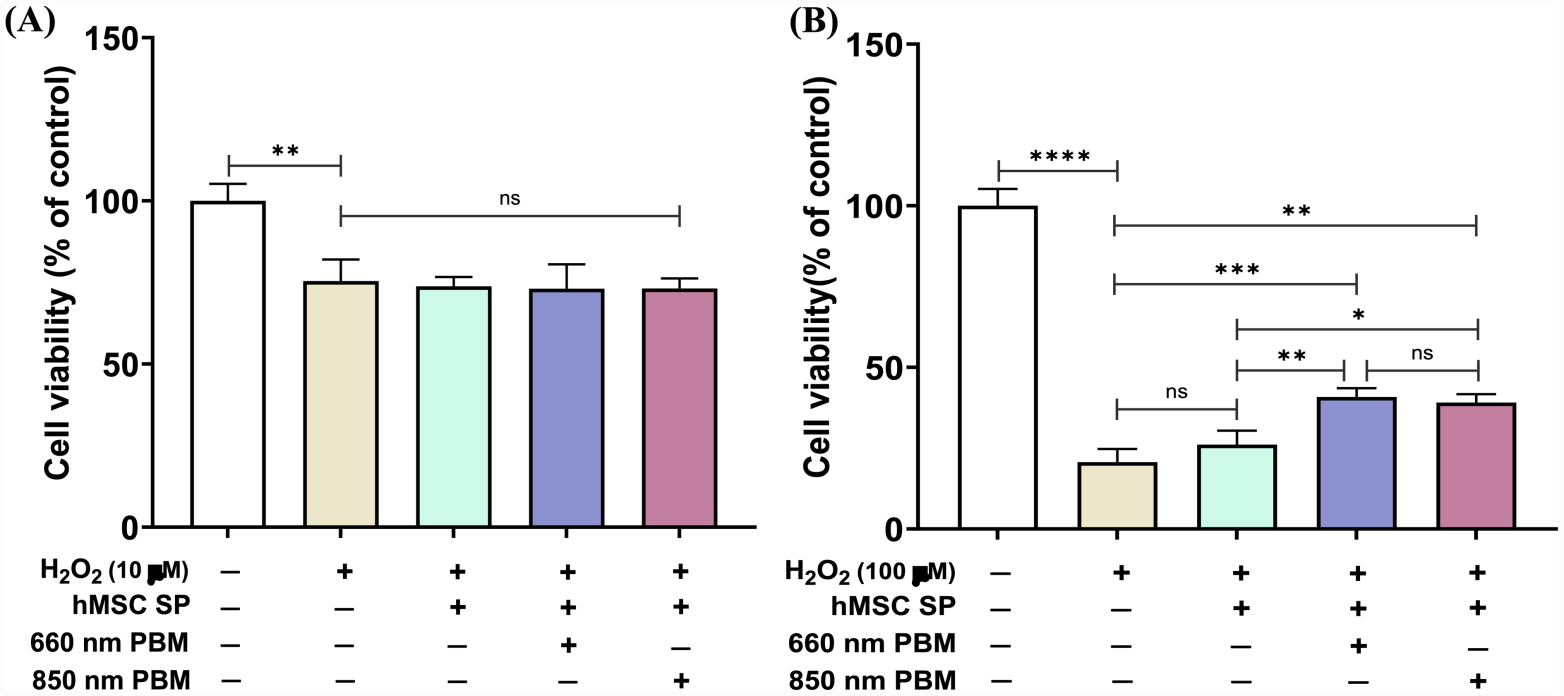
Effects of PBM-treated hMSC spheroids on neuronal viability assessed by MTT assay. **(A)** Neuronal cell viability was significantly reduced following exposure to low concentrations of H₂O₂ (10 μM for 30 min); however, co-culture with hMSC spheroids did not significantly alter cell viability under these conditions (**p < 0.01). **(B)** Exposure to high concentrations of H₂O₂ (100 μM for 30 min) markedly decreased neuronal cell viability. Co-culture with PBM-treated hMSC spheroids at both 660 nm and 850 nm wavelengths significantly improved cell viability compared to neurons exposed to H₂O₂ alone or co-cultured with non-PBM-treated spheroids (**p < 0.01, ***p < 0.001, ****p < 0.0001).

H_2_O_2_ exposure represents a cytotoxic stimulus that can cause damage to neuronal cells. Exposure to 10 μM H₂O₂ resulted in a significant reduction in cell viability to approximately 75% relative to the control (*p* = 0.0016; Figure 2A). At higher H_2_O_2_ concentrations, cell viability was drastically decreased to 21% (*p* < 0.0001; Figure 2B). To investigate the neuronal regenerative potential of hMSCs in an H_2_O_2_ toxicity model, hMSC spheroids, with or without PBM preconditioning (660 nm or 850 nm), were co-incubated with primary cortical neurons exposed to H₂O₂. Under low H_2_O_2_ concentration conditions, co-incubation with various hMSC spheroids did not alter cell viability (Figure 2A). In contrast, under high H_2_O_2_ concentration conditions, co-incubation with hMSC spheroids led to an increase in cell viability. Notably, both 660 nm- and 850 nm-PBM-treated hMSC spheroids significantly improved cell viability compared to the H₂O₂-only group and the spheroid group without PBM (*p* = 0.0006 for 660 nm vs. H₂O₂ only; *p* = 0.0013 for 850 nm vs. H₂O₂ only; Figure 2B). To assess whether PBM efficacy depends on irradiation power density, we performed a power titration for each wavelength prior to spheroid application (660 nm: 5/10/20 mW/cm^2^; 850 nm: 6/12/24 mW/cm^2^; 10 min). While PBM power titration did not markedly alter viability under low oxidative stress conditions, several power settings improved viability under high H₂O₂ conditions, indicating that PBM effects on hMSC spheroid-mediated neuroprotection are parameter-sensitive (Supplementary Figure 1).

### Observation of axonal loss and re-sprouting, and their modulation by hMSC spheroids, with or without PBM treatment, under H_2_O_2_-induced toxicity

**Figure 3:**
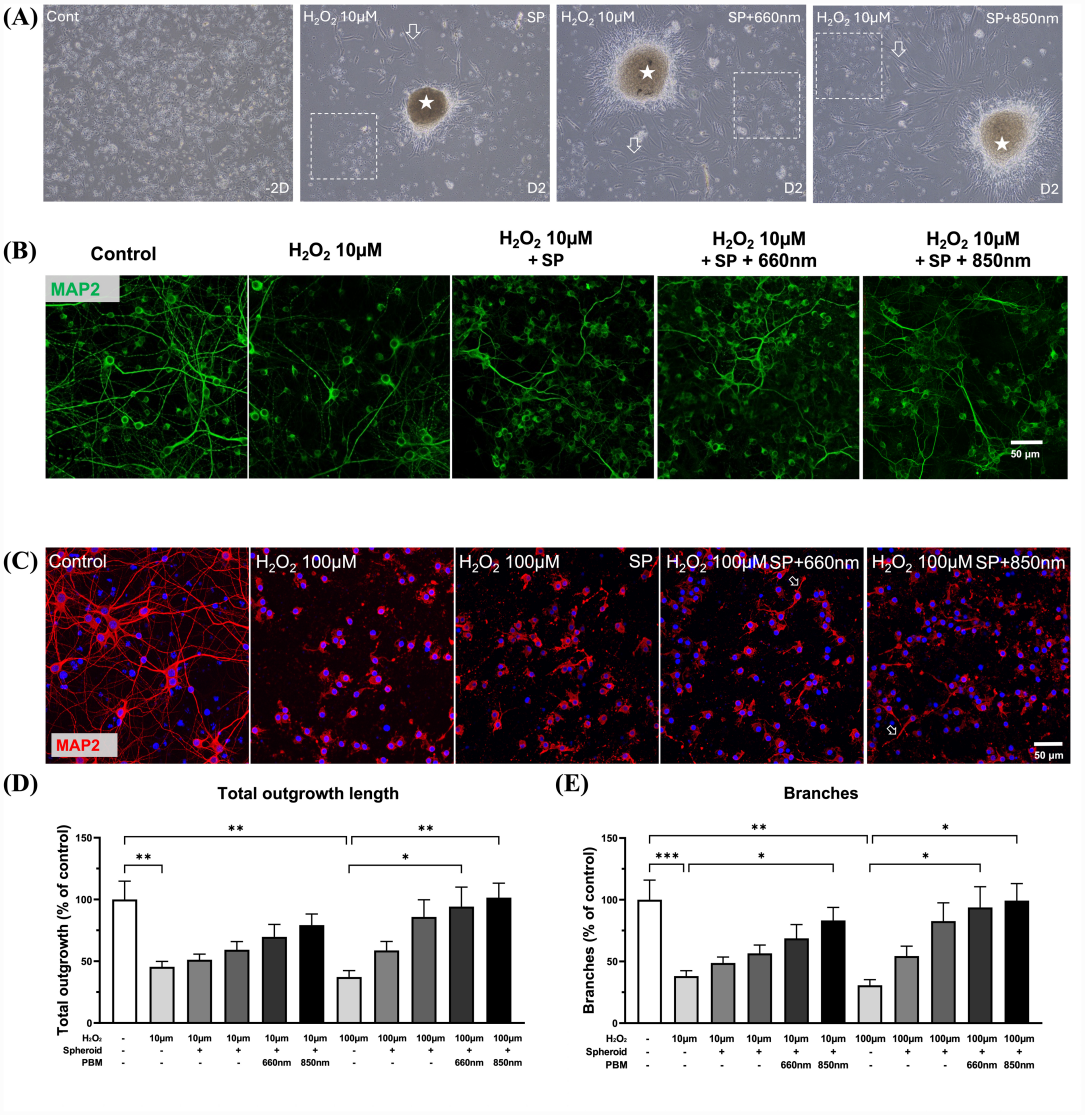
Morphological analysis of axonal damage and re-sprouting. **(A)** Representative light microscopic images of primary cortical neuron cultures under four experimental conditions are shown. Control cultures exhibited high neuronal density prior to low-concentration H₂O₂ exposure (10 μM for 30 min, day −2). The remaining groups show cultures following H₂O₂ treatment and subsequent co-culture with hMSC spheroids (white asterisks), with or without PBM treatment (660 nm or 850 nm). hMSCs dissociated from spheroids and spread into the surrounding area (open arrowheads). Dotted rectangles indicate regions analyzed for neuronal density that were not in direct contact with hMSCs. **(B)** Epifluorescence images of MAP2-stained neurons following low-concentration H₂O₂ exposure (10 μM). **(C)** Epifluorescence images of MAP2-stained neurons following high-concentration H₂O₂ exposure (100 μM). Open arrowheads indicate axonal sprouts. **(D,E)** Quantitative analysis of total axonal outgrowth length **(D)** and axonal branch density **(E)**. Under low-concentration H₂O₂ conditions (10 μM), total outgrowth length and branch density were reduced to 45.48 ± 4.35% and 38.19 ± 4.34% of control levels, respectively. Under high-concentration H₂O₂ conditions (100 μM), total outgrowth length and branch density were further reduced to 37.28 ± 5.13% and 30.67 ± 4.58% of control levels, respectively. Co-culture with PBM-treated hMSC spheroids significantly improved axonal parameters compared to the H₂O₂-only group. In particular, total outgrowth length increased to 94.19 ± 15.79% (660 nm) and 101.49 ± 11.71% (850 nm) of control levels, while branch density increased to 93.81 ± 16.76% (660 nm) and 99.36 ± 13.77% (850 nm) of control levels, under high-concentration H₂O₂ conditions, indicating substantial recovery. Under low H₂O₂ conditions (10 μM), axonal parameters in the 850 nm PBM-treated group were not significantly different from the control group, whereas the 660 nm PBM-treated group remained significantly lower than control group. Under high H₂O₂ conditions (100 μM), neurite outgrowth and branch density in the 850 nm PBM-treated group were not significantly different from the control group, indicating near-complete recovery. Data are presented as mean ± SEM (*p < 0.05, **p < 0.01, ***p < 0.001).

Primary cortical neurons were exposed to cytotoxic H_2_O_2_ (10 or 100 μM for 30 min) and subsequently co-cultivated with spheroids, with or without PBM. On day 1, samples were collected for cell viability assessment and epifluorescence analysis. After co-cultivation, hMSCs spread out, and some were in contact with primary cortical neurons (Figure 3A). For microscopic analyses, areas not in contact with hMSCs were assessed. (Figure 3A). After H_2_O_2_ exposure, even at low concentrations, the number of cells observed in light microscopy (Figure 3A) and the number of neuronal cells (MAP2) in epifluorescence analysis (Figure 3B) were significantly decreased. Epifluorescence analysis showed that the number of MAP2-expressing cells after high-concentration H_2_O_2_ exposure was also significantly reduced (Figure 3C). To evaluate the axonal re-sprouting potential of hMSCs in an H_2_O_2_ toxicity model, the total outgrowth length and rate of branching were evaluated. Across all H_2_O_2_ treatment conditions, axonal sprouting was decreased and shortened (Figures 3B,C). Under low-concentration H₂O₂ conditions, sprouting frequency (branches) was reduced to 38.19 ± 4.34% of control levels (*p* = 0.0008), and total outgrowth length decreased to 45.48 ± 4.35% of control levels (*p* = 0.0015). Under high H₂O₂ concentration conditions, sprouting frequency (branches) was reduced to 30.67 ± 4.58% of control levels (*p* = 0.0019), and total outgrowth length decreased to 37.28 ± 5.13% of control levels (*p* = 0.0023; Figures 3D,E). As shown in representative images after low H_2_O_2_ exposure, the frequency of axonal branching was relatively higher in hMSC spheroid co-cultures, especially those involving PBM-treated hMSC spheroids (Figure 3B). There was a statistically higher sprouting frequency in the 850 nm PBM-treated hMSC spheroid co-incubation group (83.25 ± 10.49% of control, *p* = 0.0263) compared to the low-concentration H_2_O_2_-only group (Figure 3E). Under high H_2_O_2_ concentration conditions, PBM-treated hMSC spheroids showed restoration of neurite parameters. Specifically, total outgrowth length increased to 94.19 ± 15.79% (660 nm, *p* = 0.0148) and 101.49 ± 11.71% (850 nm, *p* = 0.0083) of control levels (Figure 3D), while branching frequency increased to 93.81 ± 16.76% (660 nm, *p* = 0.0122) and 99.36 ± 13.77% (850 nm, *p* = 0.0102) of control levels (Figure 3E), compared to the H_2_O_2_-only group. To distinguish between direct cell–cell interactions and paracrine-mediated effects, we compared a direct co-culture of hMSC spheroids with primary cortical neurons to an indirect co-culture system using 0.4 μm Transwell inserts. The inserts physically prevent cell contact while allowing the diffusion of secreted factors. Quantitative analysis of MAP2-positive neurite outgrowth and branch density revealed that neuronal survival and axonal regeneration were comparably enhanced in both direct and Transwell co-culture conditions (Supplementary Figure 2). These results indicate that the neuroprotective and neuritogenic effects of PBM-treated hMSC spheroids are predominantly mediated by paracrine signaling rather than direct cell–cell contact.

## Discussion

PBM has emerged as a promising adjunct to enhance the therapeutic efficacy of hMSCs in neural regeneration. A growing body of evidence indicates that PBM influences stem cell behavior through the modulation of mitochondrial activity, redox signaling, and gene expression (Chang, 2020; Chang et al., 2019). Specifically, PBM has been shown to improve stem cell viability, modulate differentiation pathways, and stimulate the secretion of biologically active paracrine factors that support tissue repair (Chang and Lee, 2021; Chang, 2020). Mitochondrial chromophores, particularly cytochrome c oxidase, absorb light in the red to near-infrared spectrum, enhancing adenosine triphosphate (ATP) production while reducing oxidative stress (Perry et al., 2018; Chen et al., 2019; Moore, 2020). The selection of PBM wavelengths within this spectral range is therefore biologically relevant and has been widely adopted in both preclinical and clinical studies. In addition to the effects of light itself, the use of 3D hMSC spheroids provides an additional layer of therapeutic enhancement. Compared to traditional 2D monolayer cultures, hMSC spheroids exhibit greater stemness, improved secretion of growth factors, and increased survival under stress conditions. These effects are largely attributed to enhanced cell–cell and cell–matrix interactions within the 3D structure, as well as the formation of physiologic gradients that simulate the *in vivo* environment. The hypoxic and mechanical cues within spheroids are known to upregulate genes associated with angiogenesis, immunomodulation, and neuroprotection (Li et al., 2021; Hill et al., 2021). Importantly, the combination of PBM with hMSC spheroids may result in additive or synergistic effects, enhancing both the intrinsic regenerative capacity of the cells and their responsiveness to injury cues.

In the present study, hMSC spheroids were preconditioned with PBM at different wavelengths and applied to an oxidative injury model using primary rat cortical neurons exposed to hydrogen peroxide (H₂O₂). As expected, H₂O₂ caused significant neuronal cell death and disrupted axonal morphology. However, co-incubation with PBM-treated hMSC spheroids significantly improved neuronal viability and promoted axonal re-sprouting. Notably, spheroids preconditioned with 850 nm light demonstrated the most robust neuroprotective effects, suggesting wavelength-dependent biological responses. The comparison between 660 nm and 850 nm wavelengths was intentionally chosen to represent the red and near-infrared regions most commonly used in PBM, allowing the evaluation of potential wavelength-specific differences in cellular activation, tissue penetration, and regenerative efficacy. This observation is consistent with previous findings showing that high wavelength light provides optimal tissue penetration and is most effective in activating cytochrome c oxidase, thus promoting mitochondrial respiration and reducing intracellular ROS levels (Chen et al., 2019).

The beneficial effects observed in this study may involve enhanced metabolic support and paracrine signaling; however, it should be noted that the present study was not designed to delineate specific intracellular signaling pathways. PBM has been widely reported to influence mitochondrial bioenergetics and redox balance in various cell types, including mesenchymal stem cells, primarily through interactions with mitochondrial chromophores such as cytochrome c oxidase. Consistent with these previous observations, PBM-treated hMSC spheroids in our study exhibited enhanced secretion of neurotrophic and angiogenic factors. Importantly, we experimentally confirmed increased levels of the BDNF, NGF, and VEGF in PBM-treated hMSC spheroids using ELISA, providing direct evidence of enhanced paracrine output rather than downstream signaling activation. These secreted factors are well known to support neuronal survival, axonal regeneration, synaptic plasticity, and local angiogenesis, thereby creating a permissive microenvironment for neural repair (Monika and Anna, 2019; Liechti et al., 2017). While the upstream molecular regulators governing PBM-induced changes in hMSC spheroid function were not directly examined in this study, the observed functional outcomes strongly support a paracrine-mediated neuroprotective mechanism. Transwell-based co-culture experiments further support a paracrine-dominant mechanism, as comparable neuroprotective and neuritogenic effects were observed in the absence of direct cell–cell contact. While direct physical interactions between hMSCs and neurons may contribute to localized effects *in vivo*, our data indicate that secreted factors from PBM-preconditioned hMSC spheroids are sufficient to drive neuronal survival and axonal regeneration under oxidative stress conditions.

Despite these promising results, it should be emphasized that the present study was designed as an *in vitro* proof-of-concept investigation, focusing specifically on oxidative stress-mediated neuronal damage. The H₂O₂-induced injury model was intentionally used as a controlled and reproducible system to isolate the effects of oxidative stress and to evaluate the paracrine-mediated neuroprotective potential of PBM-preconditioned hMSC spheroids under defined conditions. Although the results are promising, several limitations must be addressed. First, the *in vitro* H₂O₂ model, while convenient and reproducible, does not fully capture the complexity of *in vivo* neural injury, which may involve excitotoxicity, inflammation, and blood–brain barrier disruption. Second, although the main experiments compared two clinically relevant wavelengths at fixed irradiation settings, comprehensive PBM optimization (e.g., varying duration, repetition cycles, and treatment frequency) was beyond the scope of this proof-of-concept study. To partially address parameter dependence, we additionally performed a power density titration for each wavelength and observed power-dependent trends in neuroprotection under high oxidative stress (Supplementary Figure 1); nevertheless, further systematic optimization will be required for translational protocol development. Third, although this study demonstrates enhanced neurotrophic factor secretion following PBM treatment, it does not identify the specific intracellular signaling pathways responsible for these changes. Future studies incorporating transcriptomic, proteomic, or pathway-specific analyses will be necessary to elucidate the molecular mechanisms underlying PBM-mediated modulation of hMSC spheroids. In addition, although immunocytochemical analyses were performed to assess cellular and neuronal characteristics, gene expression analyses of extracellular matrix (ECM)-related markers were not included in the present study. Given the critical role of ECM components in neurite outgrowth, synaptic stability, and stem cell-mediated regeneration, future studies incorporating quantitative PCR or transcriptomic approaches would provide valuable insight into ECM remodeling induced by PBM-preconditioned hMSC spheroids. Fourth, the present study is limited by the absence of *in vivo* validation, as all findings were derived from an *in vitro* co-culture system. This experimental design was intentionally chosen to establish a controlled proof-of-concept platform that enables systematic evaluation of PBM-preconditioned hMSC spheroids and their paracrine-mediated effects on injured neurons. Although the observed *in vitro* neuroprotective and regenerative effects are encouraging, they do not directly predict therapeutic efficacy *in vivo*. Future studies employing relevant animal models of neural injury, such as spinal cord injury or ischemic stroke, will be essential to validate the translational potential of this approach (He et al., 2019). In addition, hMSCs used in this study were derived from a single donor, which may limit the generalizability of the findings due to donor-to-donor variability in proliferation capacity and secretory profiles. However, the use of a single donor allowed controlled within-sample comparisons of PBM effects and minimized confounding biological variability. Future studies incorporating hMSCs from multiple donors of different ages and backgrounds will be necessary to validate the reproducibility and robustness of the observed effects.

In conclusion, this study supports the use of PBM-preconditioned hMSC spheroids as a viable therapeutic strategy for neural repair under oxidative stress conditions. The results highlight the additive benefits of combining PBM with 3D spheroid culture, particularly through enhanced mitochondrial metabolism and increased secretion of key neuroregenerative factors. The observed wavelength-specific effects, especially the superiority of 850 nm light, emphasize the importance of carefully selecting PBM parameters to maximize efficacy. These findings align with broader efforts in regenerative neuroscience to develop stem cell-based interventions for conditions such as stroke, spinal cord injury, and neurodegenerative diseases (Lunken et al., 2023). Future research should focus on elucidating the molecular mechanisms involved, optimizing PBM protocols, and validating these approaches in preclinical models to facilitate clinical translation.

## Data Availability

The raw data supporting the conclusions of this article will be made available by the authors, without undue reservation.
