# Supplementary material for: Photobiomodulation (PBM) irradiation enhances the therapeutic potential of hMSC spheroids for neural repair
Source: Front Cell Neurosci. 2026 Jan 29;20:1728579. doi: 10.3389/fncel.2026.1728579 (PMC12894004; doi:10.3389/fncel.2026.1728579)
Supplement: Supplementary file 1 [file Presentation_1.PPTX]

## Slide 1
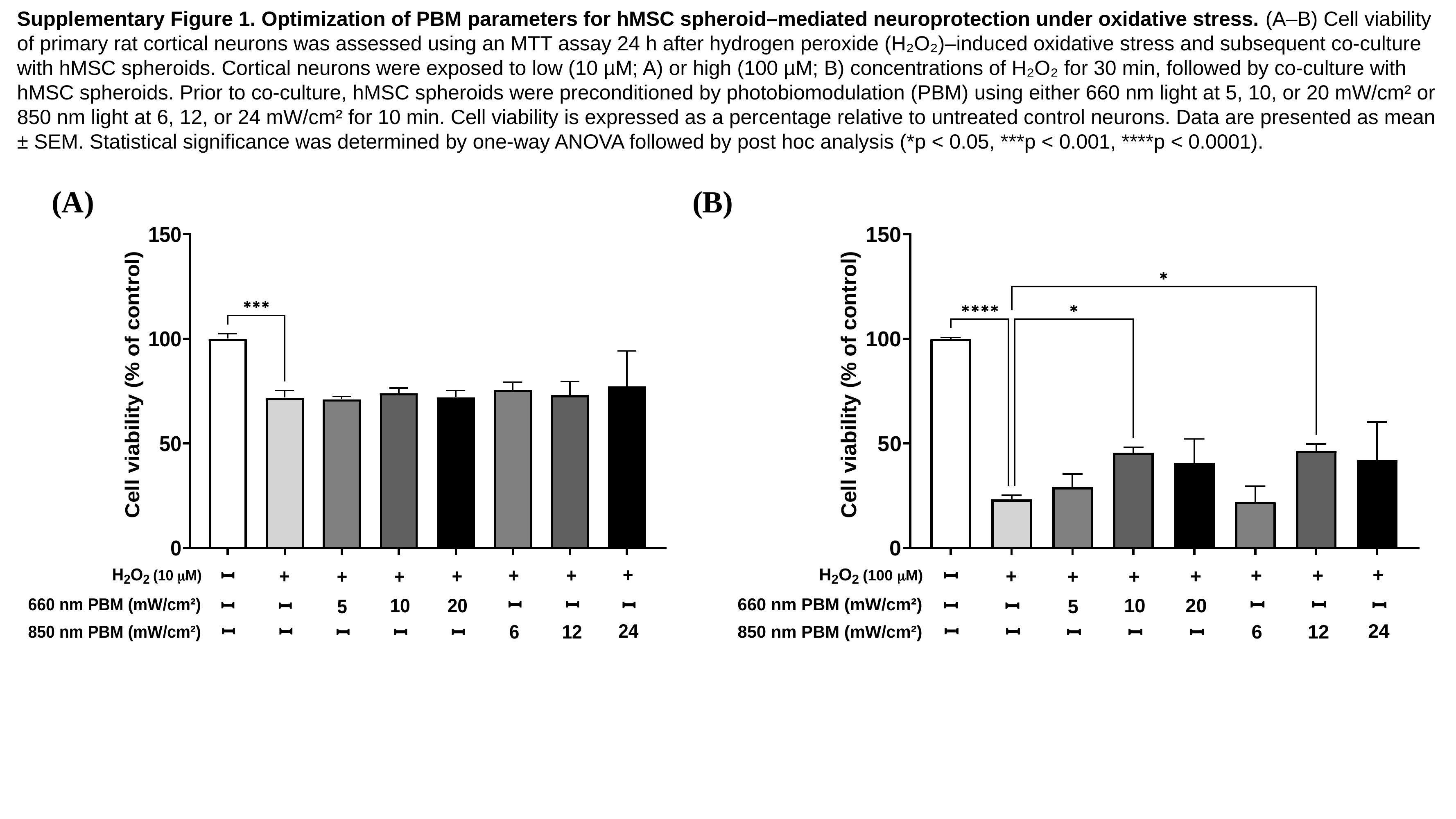

Supplementary Figure 1. Optimization of PBM parameters for hMSC spheroid–mediated neuroprotection under oxidative stress. (A–B) Cell viability of primary rat cortical neurons was assessed using an MTT assay 24 h after hydrogen peroxide (H₂O₂)–induced oxidative stress and subsequent co-culture with hMSC spheroids. Cortical neurons were exposed to low (10 µM; A) or high (100 µM; B) concentrations of H₂O₂ for 30 min, followed by co-culture with hMSC spheroids. Prior to co-culture, hMSC spheroids were preconditioned by photobiomodulation (PBM) using either 660 nm light at 5, 10, or 20 mW/cm² or 850 nm light at 6, 12, or 24 mW/cm² for 10 min. Cell viability is expressed as a percentage relative to untreated control neurons. Data are presented as mean ± SEM. Statistical significance was determined by one-way ANOVA followed by post hoc analysis (*p < 0.05, ***p < 0.001, ****p < 0.0001).
(A)
(B)

## Slide 2
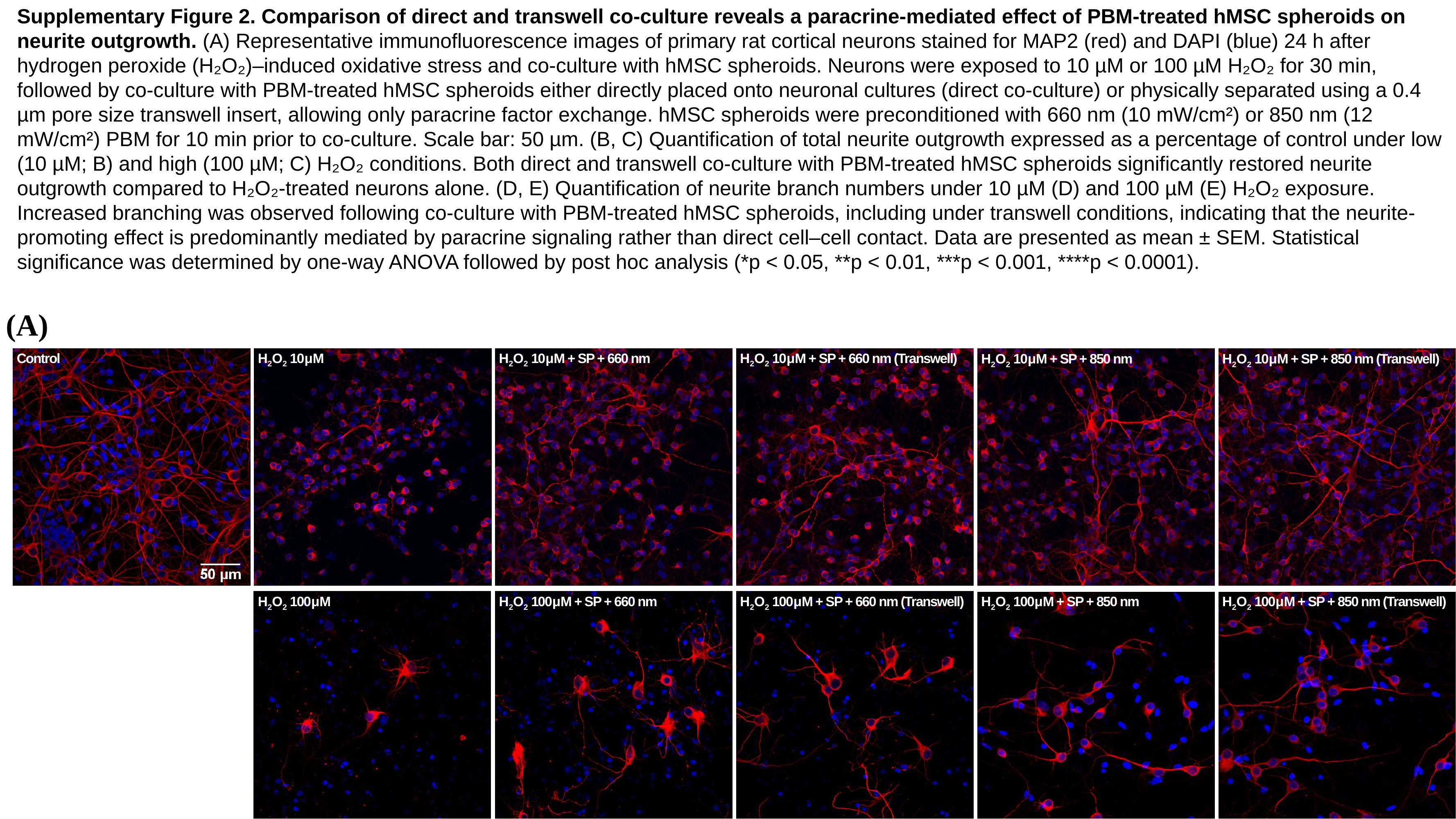

Supplementary Figure 2. Comparison of direct and transwell co-culture reveals a paracrine-mediated effect of PBM-treated hMSC spheroids on neurite outgrowth. (A) Representative immunofluorescence images of primary rat cortical neurons stained for MAP2 (red) and DAPI (blue) 24 h after hydrogen peroxide (H₂O₂)–induced oxidative stress and co-culture with hMSC spheroids. Neurons were exposed to 10 µM or 100 µM H₂O₂ for 30 min, followed by co-culture with PBM-treated hMSC spheroids either directly placed onto neuronal cultures (direct co-culture) or physically separated using a 0.4 µm pore size transwell insert, allowing only paracrine factor exchange. hMSC spheroids were preconditioned with 660 nm (10 mW/cm²) or 850 nm (12 mW/cm²) PBM for 10 min prior to co-culture. Scale bar: 50 µm. (B, C) Quantification of total neurite outgrowth expressed as a percentage of control under low (10 µM; B) and high (100 µM; C) H₂O₂ conditions. Both direct and transwell co-culture with PBM-treated hMSC spheroids significantly restored neurite outgrowth compared to H₂O₂-treated neurons alone. (D, E) Quantification of neurite branch numbers under 10 µM (D) and 100 µM (E) H₂O₂ exposure. Increased branching was observed following co-culture with PBM-treated hMSC spheroids, including under transwell conditions, indicating that the neurite-promoting effect is predominantly mediated by paracrine signaling rather than direct cell–cell contact. Data are presented as mean ± SEM. Statistical significance was determined by one-way ANOVA followed by post hoc analysis (*p < 0.05, **p < 0.01, ***p < 0.001, ****p < 0.0001).
(A)
Control
H2O2 10μM
H2O2 10μM + SP + 660 nm
H2O2 10μM + SP + 660 nm (Transwell)
H2O2 10μM + SP + 850 nm
H2O2 10μM + SP + 850 nm (Transwell)
H2O2 100μM
H2O2 100μM + SP + 660 nm
H2O2 100μM + SP + 660 nm (Transwell)
H2O2 100μM + SP + 850 nm
H2O2 100μM + SP + 850 nm (Transwell)
(B)
(C)
(D)
(E)
